# Autophagy in Skeletal Muscle Homeostasis and in Muscular Dystrophies

**DOI:** 10.3390/cells1030325

**Published:** 2012-07-25

**Authors:** Paolo Grumati, Paolo Bonaldo

**Affiliations:** Department of Biomedical Sciences, University of Padova, Viale Giuseppe Colombo 3, Padova 35131, Italy

**Keywords:** autophagy, skeletal muscle, dystrophy

## Abstract

Skeletal muscles are the agent of motion and one of the most important tissues responsible for the control of metabolism. The maintenance of muscle homeostasis is finely regulated by the balance between catabolic and anabolic process. Macroautophagy (or autophagy) is a catabolic process that provides the degradation of protein aggregation and damaged organelles through the fusion between autophagosomes and lysosomes. Proper regulation of the autophagy flux is fundamental for the homeostasis of skeletal muscles during physiological situations and in response to stress. Defective as well as excessive autophagy is harmful for muscle health and has a pathogenic role in several forms of muscle diseases. This review will focus on the role of autophagy in muscle homeostasis and diseases.

## 1. Introduction

Skeletal muscles, together with bones, are the main body’s agents of the locomotion. Precise and coordinated movements are allowed by the highly organized structure of the cytosol of muscle fibers (or myofibers), the multinucleated and highly specialized cells of skeletal muscles involved in contraction. Contractile proteins are assembled into repetitive structures, the basal unit of which is the sarcomere, that are well packed into the myofiber cytosol. Myonuclei are located at the edge of the myofibers, under the basal lamina, whereas the various organelles such as mitochondria and sarcoplasmic reticulum are embedded among the myofibrils. A proper organization and function of mitochondria and sarcoplasmic reticulum is fundamental for a prompt supply of ATP and a correct release of calcium, respectively. This precise and finely regulated arrangement of contractile proteins and organelles is a peculiar feature of muscle cells. 

Skeletal muscle is a plastic tissue that promptly responds and adapts to many different physiological conditions, such as physical exercise, loading, hormonal stimulation, and diet modification. Many different changes take place in the cytosol of myofibers during catabolic conditions: proteins are mobilized, organelles networks are reorganized to promptly supply energy needs, and also the setting of myonuclei can be modified. Moreover, strenuous physical activity, improper dietary regimens and aging lead to mechanical and metabolic damages of myofiber organelles, especially mitochondria, and contractile proteins. For example, physical exercise requires a higher level of energy, which is produced by mitochondria. Side effects of excessive physical exercise are the production of reactive oxygen species (ROS) and the mechanical damage of mitochondria [1,2]. During aging the protein turnover is slowed down, therefore it is easier to accumulate aggregates of dysfunctional proteins inside the cytosol of different cells, including myofibers [3]. For these reasons, a highly dynamic tissue such as skeletal muscle requires a rapid and efficient system for the removal of altered organelles, the elimination of protein aggregates, and the disposal of toxic products that may lead to cell death, thus allowing for the proper contraction of sarcomeres. On the other hand, protein degradation in skeletal muscles needs to be finely regulated, since an excessive induction of protein degradation would be extremely detrimental for the homeostasis of the entire body [2].

The two major proteolytic systems in muscle are the ubiquitin-proteasome and the autophagy-lysosome pathways. The proteasome system requires the transcription of the two ubiquitin ligases atrogin-1 and MuRF1 and the ubiquitination of the substrates [4]. Therefore, the ubiquitin-proteasome system can provide the rapid elimination of single proteins or small aggregates. Conversely, the autophagic system is able to degrade entire organelles and large proteins aggregates. In the autophagy-lysosome system, double-membrane vesicles named autophagosomes are able to engulf a portion of the cytosol and fuse with lysosomes, where their content is completely degraded by lytic enzymes. The lysosomal machinery provides the recovery of amino acids, which supply the energy needs of muscle cells [5,6,7]. Insight into the role of autophagy in skeletal muscle was delayed by the difficulties to detect the autophagosomes *in vivo*, within the myofiber structure. The development of new biochemical and imaging tools, able to follow autophagy flux, from autophagosomes formation to their fusion with lysosomes, greatly improved the characterization of autophagy in muscle homeostasis and under pathological conditions [8,9]. The autophagy flux can be biochemicaly monitored following LC3 lipidation and p62 degradation. LC3 is the mammalian homolog of the yeast Atg8 gene, which is lipidated when recruited for the double-membrane commitment and growth [10]. p62 (SQSTM-1) is a polyubiquitin-binding protein involved in the proteasome system and that can either reside free in the cytosol and nucleus or occur within autophagosomes and lysosomes [9,10,11]. p62 is degradated by lityc enzymes after the fusion of the autophagosome with the lysosome [9,10]. The first imaging tool to analyze the autophagosome structures was electron microscopy [12]. Only the high image resolution of this technique allows to observe the double membranes of the autophagosomes and their content. Anyway, this tool permits the analysis of a small area of the tissue and its usefulness is limited [9,10,12]. A great improvement in the analysis of autophagy *in vivo* was provided by the generation of the GFP-LC3 transgenic mouse. This animal model allows easy detection of autophagosomes by simply monitoring the presence of bright GFP-positive puncta inside the myofibrils and beneath the plasma membrane of the myofibers. This tool has allowed to investigate the activation of autophagy in skeletal muscles with different contents of slow and fast-twitching myofibers and in response to stimuli such as fasting. For example, in the fast-twiching *extensor digitorum longus* muscle few GFP-LC3 dots were observed before starvation, while many small GFP-LC3 puncta appeared between myofibrils and in the perinuclear regions after 24 h starvation. Conversely, in the slow-twitching *soleus* muscle, autophagic puncta were almost absent in standard condition and scarcely induced after 24 h starvation [13]. Although the function of autophagy in skeletal muscle is not yet fully understood, it is becoming clear that autophagy may play both beneficial and detrimental effects, depending on the specific tissue condition and the level of activation of the autophagic process. Thus, autophagy can contribute to muscle loss during atrophy [14] and sarcopenia [15], but on the other side a correct autophagy flux is fundamental for myofiber survival [16,17].

## 2. Autophagy in Muscle Homeostasis

Due to the scarcity of tools and the intrinsic difficulty to analyze a tissue such as skeletal muscle, the physiological role of autophagy in muscles is still unclear. The autophagic flux was found to be increased during certain catabolic conditions, such as fasting [7,8,13], atrophy [18], and denervation [19], thus contributing to protein breakdown. Food deprivation is one of the strongest stimuli known to induce autophagy in muscle. Indeed skeletal muscle, after the liver, is the most responsive tissue to autophagy activation during food deprivation. Since muscles are the biggest reserve of amino acids in the body, during fasting autophagy has the vital role to maintain the amino acid pool by digesting muscular protein and organelles [13]. In mammalian cells, mTORC1, which consists of mTOR and Raptor, is the nutrient sensor that negatively regulates autophagy. During atrophy, protein breakdown is mediated by atrogenes, which are under the forkhead box O (FoxO) transcription factors control [20], and activation of autophagy seems to aggravate muscle loss during atrophy. *In vivo* and *in vitro* studies demonstrated that several genes coding for components of the autophagic machinery, such as LC3, GABARAP, Vps34, Atg12 and Bnip3, are controlled by FoxO3 transcription factor [14,21]. FoxO3 is able to regulate independently the ubiquitin-proteasome system and the autophagy-lysosome machinery *in vivo* and *in vitro* [14,21]. Denervation is also able to induce autophagy in skeletal muscle, although at a slower rate than fasting. This effect is mediated by RUNX1, a transcription factor upregulated during autophagy; the lack of RUNX1 results in excessive autophagic flux in denervated muscle and leads to atrophy [22].

On the other side, the feature of aged protein accumulation, abnormal and dysfunctional mitochondria and dilated sarcoplasmic reticulum are typical feature of several muscle diseases, suggesting a deleterious scenario in the case of an impairment of autophagy flux. For instance, protein aggregates positive for ubiquitin and p62/SQSTM1 protein have been found inside myofibers of patients affected by sporadic inclusion body myositis [23]. Moreover, accumulation of dysfunctional mitochondria and dilated sarcoplasmic reticulum was described in two inherited muscle diseases, Bethlem myopathy and Ullrich congenital muscular dystrophy [24]. 

**Figure 1:**
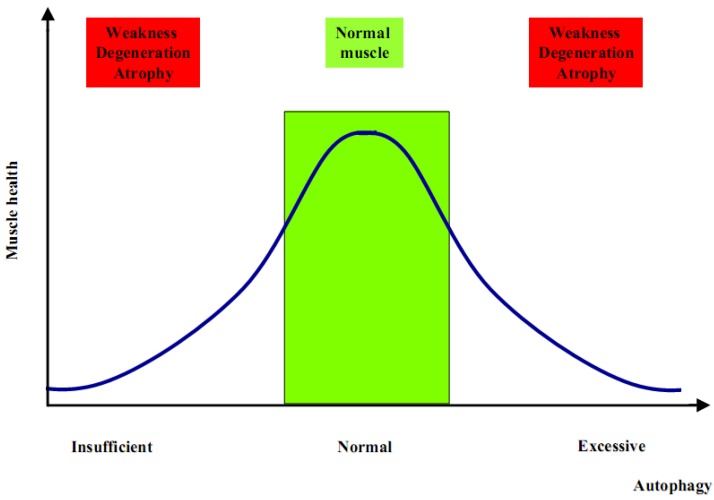
Insufficient or excessive autophagy is detrimental for muscle homeostasis.

The generation of Atg5 and Atg7 muscle-specific knockout mice has been the first step to better clarify the role of autophagy in muscle physiology. These knockout models showed that suppression of autophagy is not beneficial for the global muscle homeostasis. Indeed, both models display muscle weakness and atrophy [25,26], with also a significant reduction of the global body weight, which is therefore strictly correlated with the important loss of muscle tissue due to an atrophic condition [26,27,28]. Although these animal models have improved our knowledge about autophagy and skeletal muscle, many aspects remain to be clarified. Increased autophagy is critical during catabolic conditions such as fasting and denervation, but at the same time it is as much detrimental when completely blocked. These two opposite conditions determine an unhealthy result in terms of muscle homeostasis. Therefore, an unbalanced autophagy flux is highly detrimental for muscle, as too much induces atrophy whereas too little leads to muscle weakness and degeneration (Figure 1). One difference in these two opposite scenarios is the timing. Muscle wasting associated to autophagy inhibition becomes evident and symptomatic only after a number of altered proteins and dysfunctional organelles are accumulated within the myofiber, thereby interfering with the normal physiological functions. Such a pathological condition becomes evident and phenotypically significant only after some months or even years. On the contrary, the excessive increase of autophagy flux is able to induce a rapid loss of muscle mass, within days or weeks [2]. Although the proper balance of the autophagic flux seems to be the key point for muscle health, there are still many open questions about the mechanisms that regulate autophagy in skeletal muscles. Addressing these questions is important not only for understanding the mechanisms underlying muscle homeostasis in physiological conditions, but also for muscle disorders since a number of findings indicate that alterations of autophagy are involved in the pathogenesis of several myopathies and dystrophies. 

Beside different pathological conditions of activation or impairment of the autophagic flux, modifications of the autophagy-lysosomal system also occur in different tissues of healthy subjects during certain physiological processes, such as aging. Multiple reports indicate that Atg proteins, as well as other proteins required for autophagy induction, have reduced expression in aged tissues and that autophagy diminishes during aging [3]. This applies, for instance, to normal human brain aging (in which Atg5, Atg7, and Beclin 1 are down-regulated) [29], insulin resistance and metabolic syndrome [30], or osteoarthritis (in which Ulk1, Beclin 1, and LC3 are down-regulated) [31]. Though still quite phenomenological and correlative, these findings suggest that a weakened state of the autophagic response may contribute to the aging phenotype. Concerning skeletal muscles, there are only few reports about the involvement of autophagy in muscular changes due to the aging. Studies in rat muscles suggested an age-related decline in autophagic degradation and a concomitant age-related increase in oxidative damage and apoptosis, which both correlate negatively with autophagy. Moreover, a constant autophagic stimulus like caloric restriction seems to ameliorate the physiological state of muscles [32,33]. In drosophila, muscle aging is characterized by the progressive accumulation of protein aggregates that associates with impaired muscle function. Several data indicate that the molecular mechanisms of protein quality control are responsible for the age-related muscle weakness during aging. In particular, increased activity of the FOXO/4E-BP axis is sufficient to delay the accumulation of protein aggregates and to preserve muscle function, an effect that is mediated at least in part by promoting the basal activity of the autophagy-lysosome system. [34]. 

Several reports proposed how a particular dietary regimen is appropriate to stimulate autophagy and to prevent some common diseases associated with the aging process and sedentary life [3,32]. Moreover, as well known, physical exercise is a healthy habit. It strengthens muscles, helps to keep body weight under control, protects against cancer, diabetes and age-related muscle loss (or sarcopenia). Nevertheless, the cellular and molecular mechanisms underlying the beneficial effects of exercise are still poorly understood. Some recent studies demonstrated how physical exercise is useful to induce autophagy in muscle and how this mechanism is an interesting way to explain the positive effect of physical activity [35,36,37,38]. Although in these studies the effects of training were limited to running exercise in mice, it is evident that autophagy is strongly induced after an acute run on the treadmill as well as after an endurance exercise. Physical activity represents a major stress condition for skeletal muscles, which need a huge amount of energy to supply contraction. Moreover, during exercise contractile proteins and organelles, such as mitochondria, are subjected to an intense load of work and this may lead to their exhaustion and, in the case of mitochondria, to the production of toxic substances and catabolites like ROS. The autophagic machinery represents a convenient and efficient way to satisfy the increased energetic needs and to eliminate the dysfunctional organelles. The autophagosomes are able to selectively engulf dysfunctional organelles and after the fusion with lysosome and degradation by lytic enzymes, amino acids can be recycled as a new source of energy [39]. 

In a recent work, we reported the first evidence of the importance of the autophagy flux during physical exercise. In this work, mice were subjected to two different protocols of locomotory activity, an endurance spontaneous training on running wheels and a short but forced exercise on treadmill. After three months of spontaneous exercise on running wheels, there were no obvious evidences of LC3 lipidation but there was a significant decrease in Akt phosphorylation. With this long-term running wheel exercise, we had an indirect evidence of autophagy induction by analyzing the response of collagen VI null (*Col6a1*^–/–^) mice, which have a dystrophic phenotype linked to an impairment of the autophagy flux [17]. After this form of exercise, *Col6a1*^–/–^ mice showed a dramatic worsening of their muscle phenotype and, unlike from wild-type controls subjected to the same treatment, their muscle still displayed a high level of Akt activity [35]. It is likely that such a long-term protocol of voluntary exercise leads to an adaptation of muscle tissue, so that induction of autophagy becomes difficult to reveal. Until now, there are no other evidences of autophagy modulation after endurance training in mouse models. Nonetheless, there is a strong indication that autophagy is activated during endurance running in humans. Indeed, two recent studies demonstrated that muscle biopsies obtained from healthy subjects after ultra-endurance exercise show a significant increase in the mRNA levels of several autophagy-related genes [36,38]. Interestingly, as in the case of mice subjected to long-term running wheel exercise, human muscles after ultra-endurance exercise also display a decrease in Akt phosphorylation and a concomitant increase in AMPK activity [38]. The second protocol we used in mice for investigating the relationship between autophagy and physical activity was acute exercise by one-hour forced running on treadmill. Interestingly, this protocol led to a dramatic activation of the autophagic flux activation in muscles of wild-type mice. Indeed, after the treadmill exercise, hindlimb muscles displayed a marked lipidation of LC3 and numerous autophagy puncta within myofibers. When comparing the histological and ultrastructural analysis of muscles of wild-type and *Col6a1*^–/–^ mice subjected in parallel to treadmill exercise, it became evident how activation of autophagy is functional in a stress situation like this. In fact, *Col6a1*^–/–^ muscles were not able to activate autophagy during the treadmill run, and as a consequence they presented a severe muscle degeneration. The most dramatic consequence of the autophagy impairment was the presence of a large amount of swollen mitochondria and of marked dilations of the sarcoplasmic reticulum in almost all the myofibers of *Col6a1*^–/–^ muscles after treadmill [35]. Probably, the impairment of autophagy is detrimental because it does not allow the supply of amino acids and the mobilization of glucose, thus determining a deleterious overload of work charged on mitochondria. Moreover, physical exercise is known to promote mitochondria biogenesis and to improve mitochondrial function, including the replacement of old mitochondria with new and energy-efficient ones. In the dystrophic *Col6a1*^–/–^ muscles, dysfunctional organelles accumulated due to the block of autophagy therefore causing increased ROS levels [40] and myofiber damage.

Recently, He *et al.* confirmed our observations on autophagy induction by physical exercise, and documented that exercise-induced autophagy plays an important and previously unrecognized role in metabolism. This work showed that autophagy is triggered by exercise in tissues involved in glucose and energy metabolism, such as liver and pancreas. The authors showed that activation of autophagy contributes to the metabolic effects of long-term training as well. Using a high-fat diet model of obesity and glucose tolerance, they found that, unlike in wild-type mice, exercise failed to improve glucose tolerance in autophagy-impaired mice fed with a high-fat diet [38]. These findings identify a link between exercise and autophagy and reveal that autophagy is a key contributor to the metabolic benefits of both acute and long-term training.

Although the above studies have revealed an important link between autophagy and physical exercise, there are still several open questions. It remains unclear whether the effects of exercise on autophagy in muscle are cell- and/or tissue-autonomous, and it is also possible that exercise determines the release of cytokines that may play an important role. Further studies are needed to fully clarify the molecular mechanisms and functions of autophagy during exercise [41].

## 3. Molecular Mechanisms of Autophagy in Skeletal Muscles

During fasting most tissues, such as liver and pancreas, show a transient activation of the autophagic flux that lasts only few hours. In skeletal muscles the situation is peculiar because generation of autophagosomes generation can persist for days [13]. This suggests that different signaling pathways may control the autophagic flux during short and long periods. The initial steps of autophagy are likely regulated by post-translational protein modifications, such as phosphorylation, which regulate protein complexes and protein interactions. Instead the prolonged autophagy induction seems to require a transcriptional control in order to replenish autophagic protein mediators, such as LC3 and p62, which are degraded after autophagosomes fusion with lysosomes. The correct balance between inhibitors and activators of the system finely regulates the autophagy flux. This equilibrium determines the formation of the autophagosomes, their delivery to lysosomes, and finally the fusion between autophagosome and lysosome [1].

Although the study of the molecular mechanisms of autophagy in skeletal muscle is still in its infancy, several studies reported few autophagy suppressors and enhancers. RUNX1 is a transcription factor that is poorly expressed in innervated muscle but strongly upregulated after denervation. Muscle-specific specific ablation of RUNX1 in mice leads to a severe atrophy after denervation. The presence of a large amount of autophagic vacuoles in denervated *Runx1* null mice indicates that excessive autophagy is responsible for this severe muscle wasting. Therefore, the induction of Runx1 appears to be required in order to limit the extension of muscle wasting and to preserve the structural integrity of myofibrils in conditions such as denervation [22]. Another negative regulator of autophagy in muscle cells is the protein phosphatase Jumpy. *In vitro* studies in C2C12 cells revealed that knockdown of Jumpy leads to markedly increased levels of lipidated LC3. Jumpy acts on the nascent autophagic membranes and acts as a ‘brake’ for the autophagy machinery. This block is applied at the PI3P-dependent initiation stage of the execution phase of autophagy, and it has been shown that the balance between PI3P production (regulated by Vps34) and PI3P hydrolysis (regulated by Jumpy) determines the induction and the baseline levels of autophagy. The critical role of Jumpy in skeletal muscle is also demonstrated by the fact that mutations of Jumpy are associated with centronuclear myopathy, a human inherited muscle disease [16]. A recent work revealed that nutrient-deprivation autophagy factor-1 (NAF-1), a BCL-2-associated autophagy regulator, is required to maintain skeletal muscle homeostasis. Naf-1 knockout mice present a progressive muscle degeneration beginning at 2-3 months. Skeletal muscles display weakness and markedly decreased strength, accompanied by increased autophagy, dysregulation of calcium homeostasis and enlarged mitochondria. Thus, NAF-1, which is required for BCL-2-associated suppression of autophagy and calcium flux in the endo/sarcoplasmic reticulum, plays a critical role in skeletal muscle health [42].

The most studied inhibitor of autophagy flux in muscle is Akt. This kinase is able to regulate autophagy mainly in two different ways: a rapid mechanism through the regulation of mTOR activity, and a slower system, independent from mTOR, which requires gene transcription via FoxO3 [14,21]. The mTOR kinase is a sensor of the nutrient state of the cell and it is important for cell growth. In skeletal muscle, hypertrophy requires mTOR and mTOR inhibition blocks muscle growth or myofiber regeneration [43]. Growth factor stimulation, such as insulin and IGF-1, primarily regulates mTOR signaling through the PI3K-Akt axis. Autophagy is mainly regulated by mTORC1, which is the complex of mTOR with its partner Raptor. Akt is able to modulate mTORC1 activity directly phosphorylating mTOR or through the tuberous sclerosis complex (TSC). Akt can phosphorylate TSC2, so that TSC complex is inhibited, thereby activating the mTORC1 pathway [43,44,45]. In *S. cerevisiae*, TORC1 negatively regulates autophagy through the regulation of ATG1, which is an autophagy-initiating kinase [45,46]. ATG1 interacts with several autophagic proteins, including ATG13 and ATG17. The interaction of ATG13 and ATG17 with ATG1 is triggered by signals that induce autophagy, such as rapamycin treatment or nutrient starvation, and formation of this complex is important for ATG1 kinase activity [47]. TORC1 appears to phosphorylate ATG13 on multiple residues to disrupt the ATG1 complex [48], thereby repressing autophagy induction. TORC1 role in the regulation of autophagy is conserved in eukaryotes [46]. Several studies revealed that the ULK1 (the human homolog of ATG1) is involved in autophagy regulation [49,50] and acts downstream of mTORC1. Moreover, recent reports show that mTORC1 interacts with ULK1-Atg13-FIP200 (a mammalian functional homolog of ATG17) [51] and directly phosphorylates ULK1 kinase and Atg13 proteins [52,53,54]. The role of mTORC1 in autophagy regulation in muscle seems to be less prominent, although muscle-specific inhibition of mTORC1 complex leads to muscular dystrophy [55]. In this respect, it is interesting to consider that an mTOR-independent but Vps34-Beclin 1 dependent regulation of the autophagic flux was reported, at least in myotubes [55,56,57]. Akt is able to regulate autophagy also at the level of gene transcription. It is well know that Akt can regulate FoxO transcription factors [20], and it was recently demonstrated that FoxO3 is necessary and sufficient to regulate protein breakdown through the lysosomal system in skeletal muscles [14,58]. As discussed above, different autophagy genes, such as LC3, GABARAP, Bnip3, Vps34, and Atg12, are under FoxO3 control. Several *in vivo* loss- and gain-of-function experiments demonstrated that the BH3-only protein Bnip3 is an important regulator of autophagy. In skeletal muscle, overexpression of Bnip3 is able to induce autophagy, while its knockdown attenuates FoxO3 action [14,17,58]. The Akt-FoxO3 axis is probably a long-term mechanism for regulating autophagy. Therefore Akt can influence the autophagy flux in different ways: acting in a rapid manner by modulating mTORC1 activity, but also maintaining autophagy active during time by regulating gene transcription.

Beside suppressors of autophagy, in skeletal muscle there are also several essential molecules for autophagy induction. Some of these proteins are directly involved in the autophagy process, such as Atg5 and Atg7. Muscle-specific inactivation of these two critical autophagy-related genes in mice determines a severe muscle wasting associated with ultrastructural alterations of cytosolic organelles, such as mitochondria, and myofibers degeneration [25,26]. The most studied of such models was the muscle-specific Atg7 null mouse, which display a general atrophic condition of skeletal muscles. Physiological studies have also revealed that autophagy-deficient muscles of *Atg7*^f/f^ mice are significantly weaker than wild-type. Histological staining revealed several morphological alterations of myofibers, and besides the presence of atrophic fibers, there were clear signs of degeneration, including vacuolated fibers and centrally located nuclei. Ultrastructural analysis revealed the presence of morphologically abnormal and dysfunctional mitochondria, of sarcoplasmic reticulum dilations and of aberrant concentric membranous structures [26,27,28]. All these features are reminiscent of the phenotype found in the mouse model for collagen VI deficiency and in patients affected by muscular dystrophies linked to mutation of *COL6* genes, which display a blockade of the autophagic flux [24]. Interestingly, although the proteasome machinery is not compromised in *Atg7*^f/f^ mice, their muscles also display an accumulation of ubiquitinated proteins, thus indicating that ubiquinated proteins are also targeted to the autophagy-lysosome system.

One potent autophagy activator is AMPK, a kinase activated by AMP that senses cellular energy status to maintain energy homeostasis [59]. When nutrients are scarce, AMPK acts as a metabolic checkpoint inhibiting cellular growth. The most described mechanism by which AMPK regulates cell growth and also autophagy is through suppression of the mTORC1 pathway, by directly phosphorylating the Raptor subunit of the mTORC1 complex [60,61]. In contrast to its inhibitory phosphorylation on mTORC1, AMPK directly activates ULK1 through phosphorylation. Mass spectrometry showed that AMPK subunits are ULK1 interactors [62,63]. Moreover, two recent studies demonstrated that AMPK can directly phosphorylate ULK1 on several sites, which are different from the site regulated by mTORC1 [64,65]. Genetic studies in *C. elegans* [64] demonstrated that Atg1 is needed for the effect of AMPK on autophagy. Collectively, these studies indicate that AMPK can trigger autophagy through a double mechanism, by directly activating ULK1 and by inhibiting the suppressive effect of mTORC1 on ULK1. Many of the temporal and spatial details of the regulation of these three evolutionarily conserved interlocking nutrient-sensitive kinases (AMPK, ULK1 and mTOR) remain to be decoded [66]. Despite its important role in autophagy, AMPK contribution to the induction of the autophagic flux in skeletal muscle has been only barely investigated until now. A recent study showed that AMPK phosphorylates FoxO3 determining its translocation to the nucleus, where it induces the expression of the autophagy-related proteins LC3, GABARAPL1, and Beclin 1 in primary mouse myotubes and in the *tibialis anterior* muscle. Moreover, AMPK activation leads to the inhibition of mTORC1 and its subsequent dissociation from ULK1 complex. Interestingly, in muscle cells ULK1 was found to interact with and to be phosphorylated by AMPK, and it has been shown that ULK1 is associated with AMPK under normal conditions and dissociates from AMPK during autophagy. These findings show that AMPK activation stimulates autophagy in skeletal muscle, through its effects on the transcriptional function of FoxO3, and that AMPK takes part in the initiation of autophagosome formation by interacting with ULK1 [67]. In addition, it was reported that during physical exercise AMPK activation is required to guarantee a proper activation of autophagy [38].

Olson’s group identified histone deacetylases (HDACs) as enhancers of autophagy in skeletal muscles. Muscle-specific ablation of both HDAC1 and HDAC2 resulted in partial perinatal lethality in mice, accompanied by mitochondrial abnormalities and sarcomere degeneration. HDAC1/2 knockout mice surviving postnatally develop a progressive myopathy characterized by autophagy impairment. HDAC1 and HDAC2 were found to regulate muscle autophagy by inducing the expression of autophagy genes. Notably, HDAC1 and HDAC2 overexpression is sufficient to promote the autophagic flux. Moreover, feeding HDAC1/2 knockout mice with a high-fat diet releases the block in autophagy and prevents myopathy [68].

The Beclin 1 complex plays a key role in the formation of autophagosomes, but there are only few data in the literature concerning the involvement of the Beclin 1 complex in autophagy regulation in skeletal muscles. Beclin 1 was found to be involved in the regulation and induction of a correct autophagic flux in muscle during fasting and physical exercise [17,38]. Moreover, Beclin 1 appears to be critically involved in the impairment of the autophagic flux in muscular diseases associated with collagen VI deficiency, as demonstrated by the markedly decreased Beclin 1 protein levels in muscles from fasted *Col6a1*^–/–^ mice and in muscle biopsies from Bethlem/Ullrich patients [17]. The mechanisms regulating Beclin 1 in muscle are still unknown although it seems that, at least during fasting, increased Beclin 1 levels are more relying on protein stability than on transcription [17]. Moreover, the involvement of the other components of the Beclin 1 interactome (such as Ambra1, UVRAG, Atg14L, Rubicon, and others) in the regulation of autophagy in skeletal muscle remains largely unknown [69].

**Figure 2:**
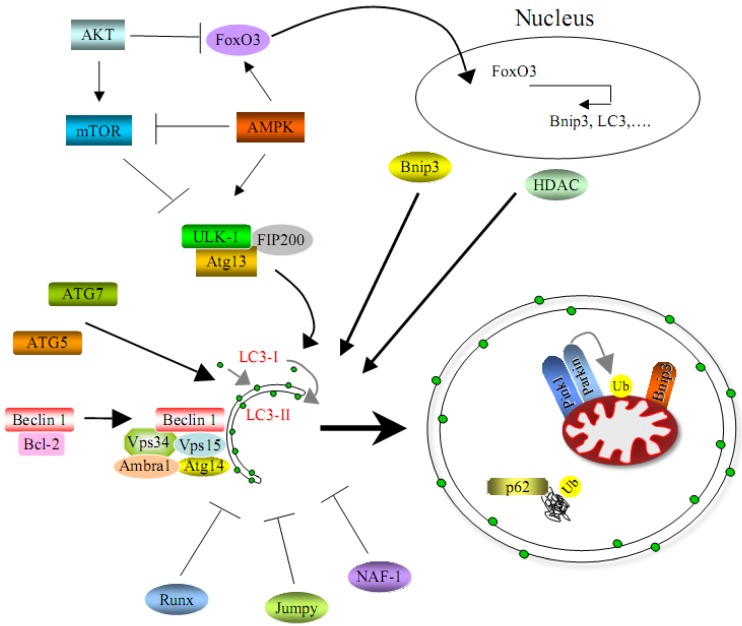
Scheme that represents positive and negative regulators of autophagy in skeletal muscles.

Altogether, a number of studies demonstrates that regulation of the autophagic machinery is highly complex and involves several signaling pathways that often crosstalk each other (Figure 2). We are still far from a detailed understanding of the autophagic process in skeletal muscles. Moreover, almost all studies reported until now for skeletal muscle concern macroautophagy, while the other processes related to the autophagy-lysosome system, such as chaperone-mediated autophagy and microautophagy, remain largely unexplored. While still relatively few animal models and technical tools for studying autophagy in the context of muscle are currently available, the development of further tools and models will likely allow to investigate in detail in the next few years the relevance of this important cellular process in skeletal muscle.

## 4. Autophagy in Congenital Muscle Dystrophies

A large number of studies carried out in the last decade has clearly established the critical role of the autophagy-lysosome system for health and disease. Many neurodegenerative conditions, for instance, can be traced back to defective autophagy, which may be the cause of the failure to clear aggregates of mutated toxic proteins. Autophagy has also been identified as crucial in cancer progression. Finally, antigen presentation, innate immune signaling and pathogen degradation may all involve autophagosome recruitment and activity [5,6,7]. While a role of autophagy has been also recognized in heart failure and cardiovascular diseases, surprisingly relatively little was known until recently about autophagy and skeletal muscle diseases, in particular muscular dystrophies.

The involvement of the autophagy-lysosome system in inherited diseases affecting muscles was limited to some disorders characterized by mutations of genes coding for proteins involved in lysosomal function, such as Pompe disease, Danon disease and X-linked myopathy with excessive autophagy [70]. Recent findings from us and other groups have demonstrated an important correlation between autophagy and congenital muscular dystrophies linked to mutation of genes coding for extracellular matrix (ECM) proteins [17,71]. The ECM is a dynamic structure that provides support and anchorage for cells, segregates tissues from one another and initiates signal transduction pathways. The ECM is composed primarily of glycoproteins, collagens and proteoglycans that are secreted and assembled locally into an organized network. An ECM is present in the mammalian embryo already from the two-cell stage and is a component of the environment of all cell types, although the composition of the ECM and the spatial relationship between cells and the ECM differ between tissues. The ECM includes the interstitial matrix and the basement membrane. The interstitial matrix is present between cells while the basement membrane is a thin sheet-like deposition of ECM that surrounds cells (e.g., muscle cells) or underlies cells (e.g., epithelial cells) [72,73]. A major role of the basement membrane is to provide a solid scaffold for the cells and to separate them from the surrounding interstitial matrix. Apart from providing tissue structure, the basement membrane is also crucial for survival and differentiation of cells. Consequently, altered basement membranes are responsible for various inherited human diseases. Particularly in skeletal muscle, mutations in genes encoding ECM proteins and their receptors are responsible for several types of muscular dystrophies and in particular congenital muscular dystrophies (CMDs) [74]. CMDs form a heterogeneous group of progressive genetic diseases, which are mostly inherited in an autosomal recessive manner. CMDs present a broad phenotypic variability, ranging from severe and lethal forms to milder types compatible with normal life span. Depending on the genetic defect, it is possible to distinguish four forms of CMDs linked to defects of ECM proteins or of their receptors, two associated with mutations in laminin α2 chain and collagen VI (MDC1A and Ullrich/Bethlem, respectively) and two associated with defects of laminin receptors (dystroglycanopathies and integrin α7 deficient congenital myopathy, respectively) [75,76,77,78,79,80,81,82].

In a pioneering study in the field of muscular dystrophies, we found that an impairment of the autophagic flux has a key pathogenic role in CMDs linked to collagen VI deficiency. This work revealed that autophagy plays a protective role against muscle fiber death in Bethlem myopathy and Ullrich congenital muscular dystrophy [17]. A failure of the autophagic machinery is responsible for the inefficient removal and the persistence of altered organelles in myofibers of collagen VI deficient (*Col6a1*^–/–^) mice. The ensuing accumulation of dysfunctional mitochondria triggers myofiber apoptosis, which in turn leads to the development of the myopathic phenotype [83]. In *Col6a1* null mice, the dramatic consequences of the autophagy inactivation are even more evident in conditions of muscle stress, such as physical exercise. When *Col6a1*^–/–^ mice are subjected to an intense work, a condition in which autophagy is required for the continuous energy need and the rapid elimination of exhausted organelles, the inefficient autophagy flux determines a massive degeneration of myofibers with a marked exacerbation of the myopathic phenotype [36]. Notably, forced activation of autophagy by dietary, genetic and pharmacological tools is able to eliminate altered organelles to restore muscle homeostasis in *Col6a1*^–/–^ mice, with recovery of the myopathic phenotype, thus opening a promising therapeutic potential for counteracting muscle atrophy and weakness in these diseases. The autophagic failure of *Col6a1* null mice strictly depends on a impairment in the “on rate” of the autophagy flux, which in turn determines a decrease in autophagosome formation and an inefficient removal of dysfunctional organelles [17]. Lack of collagen VI has a remarkable impact on molecules involved in the regulation of autophagy, with decreased protein levels of Beclin 1 and Bnip3 and persistent activation of the Akt/mTOR pathway even during starvation [17,84]. Although the molecular pathways transducing collagen VI signals from the ECM to the autophagy machinery remain to be elucidated, the Beclin 1 complex and the AKT/mTOR pathway are markedly affected by lack of collagen VI and these alterations appear to be the main cause for the autophagy inhibition. As discussed also above, it is well established that Beclin 1 is a key player in autophagy induction and autophagosome formation [69,85]. While in normal muscles Beclin 1 increases its protein levels after starvation, in *Col6a1*^–/–^ muscle Beclin 1 levels remain lower and are not induced by starvation. Interestingly, real time PCR revealed that there are no variations in Beclin 1 transcript levels between wild type and *Col6a1*^–/–^ muscles, neither in standard condition nor after fasting, suggesting that the modulation of Beclin 1 may be determined more by its protein stability and/or protein-protein interactions than at the level of transcriptional regulation. Notably, Beclin 1 protein levels are also lowered in muscle biopsies of patients affected by collagen VI diseases, and the amount of Beclin 1 seems to correlate with the severity of the phenotype, being much lower in the severe Ullrich CMD than in the milder Bethlem myopathy [17]. Besides the Beclin 1 defect, the Akt/mTOR axis seems to have an important role in the autophagic failure, since *Col6a1*^–/–^ muscles maintain a persistent Akt activity even after starvation. As discussed in detail above, Akt is able to block autophagy both through mTOR pathway inhibition and through FoxO transcription factors sequestration in the cytosol. The persistent activation of the Akt/mTOR pathway in *Col6a1*^–/–^ muscles determines a general inhibition of the autophagy machinery at the beginning of the process, during the formation of the autophagosomes. The consequent inhibition of FoxO in *Col6a1*^–/–^ muscles determines the block of the transcription of several genes, including Bnip3, which are required for the induction of autophagy in skeletal muscles [14,17]. Besides its general function in autophagy activation, Bnip3 has also an important role in the selective removal of damaged or dysfunctional mitochondria, a process known as ‘mitophagy’ [86]. It has been demonstrated that ATG32 is required for selective mitophagy in yeast [87]. In mammals, Parkin, Pink1 and Bnip3L are critical for mitophagy and their inactivation was demonstrated to be responsible for the presence of abnormal mitochondria. Parkin and Pink1 associate together to form a key quality control mechanism able to selectively recognize altered mitochondria and to target them to the lysosomal system [88,89]. On the other hand, Bnip3L links LC3, so that it directly recruits nascent autophagosomes to mitochondria [86,90]. Interestingly, we found that the autophagic failure of *Col6a1*^–/–^ muscles is also associated with a decrease of Bnip3 levels [17], suggesting that the selective removal of mitochondria by mitophagy may be also impaired in collagen VI diseases. Considering the key regulators of mitophagy, besides Bnip3, in the future it will be useful to investigate also the possible involvement of Pink1 and Parkin in the defective clearance of dysfunctional mitochondria. 

Our work in *Col6a1*^–/–^ mice and in Bethlem/Ullrich patients represented the first evidence that an impairment of the autophagic flux plays a crucial role in the pathogenesis of muscular dystrophies, thus opening new venues for therapeutic approaches and paving the way for investigating autophagy defects in other muscular dystrophies [17,91]. Indeed, a recent study revealed that the *dy^3K^/dy^3K^* mouse, which has laminin α2 deficiency and represents a model of human MDC1A, has an alteration of autophagy. Interestingly, differently from *Col6a1*^–/–^ animals, *dy^3K^/dy^3K^* mice display a general upregulation of the autophagic machinery and the inhibition of autophagy significantly improves their dystrophic phenotype [92]. The increased autophagic flux of *dy^3K^/dy^3K^* muscles is due to inactivation of Akt, which in turn determines the enhanced transcription of several autophagy genes under FoxO3 control. Moreover, mRNA levels of several lysosomal markers, such as cathepsin L and Lamp-2a, are significantly increased in *dy^3K^/dy^3K^* mouse. Similar findings were obtained in primary myoblasts and myotubes from MDC1A patients [92]. This study did not report a detailed analysis of other autophagy pathways, but considering the unphosphorylated state of Akt, it seems plausible that at least the entire mTOR pathway may be down-regulated. Notably, in a separate study the same group reported that also the proteasome system is upregulated in these mice and that its pharmacological inhibition through MG-132 partially recovers the muscle phenotype [93]. Taken together, these findings indicate that in *dy^3K^/dy^3K^* mice there is a general increase of proteolysis that involves both the autophagy-lysosome and the ubiquitin-proteasome system. 

**Figure 3:**
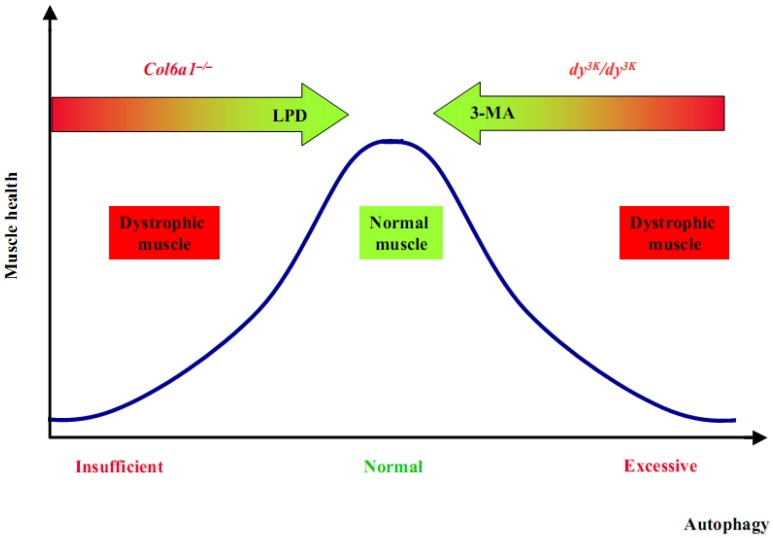
The re-establishment of a correct autophagy flux can rescue the dystrophic phenotypes.

These studies are of high interest in the field of autophagy and muscular dystrophies, since they demonstrate that an incorrect activity of autophagy plays a key pathogenic role in two of the most common forms of CMDs, both linked to deficiency of ECM proteins. How the absence of two different ECM proteins results in such opposing effects on autophagy remains completely unknown. Although much work remain to be done in order to elucidate in detail the molecular mechanisms that connect ECM to the autophagy machinery, it is evident that in both *Col6a1*^–/–^ and *dy^3K^/dy^3K^* animals it is possible to rescue the dystrophic phenotype and reestablish myofiber homeostasis by modulating autophagy (Figure 3).

## 5. Lysosome and Autophagy Crosstalk

Accumulation of autophagosomes represents the major feature of a group of muscle disorders collectively known as autophagic vacuolar myopathies [71,94]. These myopathies are characterized by mutations of genes coding for proteins involved in lysosomal function and include Pompe disease, which is due to a defect in lysosomal acid α-glucosidase, Danon disease, caused by mutations of the LAMP2 gene, and X-linked myopathy with excessive autophagy (or XMEA), which is associated to mutations of the VMA21 gene, the principal mammalian proton pump complex [94,95]. It is still unclear whether accumulation of autophagosomes in these diseases contributes to muscle damage, or conversely is a compensatory effect. Indeed, for many years the pathogenic mechanisms of autophagic vacuolar myopathies was attributed to the impairment and/or the rupture of lysosomes. However, this view was challenged by more recent works, which suggested that a massive accumulation of autophagosomes, resulting from a failure of productive autophagy, may be the major event causing myofibrillar disorganization [96]. Anyway, this hypothesis is not sufficient to completely clarify all the features of these myopathies, since the inhibition of autophagy flux in mouse model of Pompe disease does not substantially ameliorate their phenotype [97].

An important issue is the crosstalk between the autophagy pathway and the lysosomes, and how these cell machineries are mutually regulated. In the recent years, the systematic analyses of the transcription factor EB (TFEB) allowed to clarify the link between autophagy and lysosomal biogenesis [98]. TFEB was found to act as a master regulatory gene for the transcription of autophagy and lysosomal genes in response to starvation. Moreover, it controls autophagy by positively regulating autophagosome formation and autophagosome-lysosome fusion both *in vitro* and *in vivo* [99]. TFEB action is determinated by its nuclear translocation. Under standard conditions, TFEB is phosphorylated, so sequestered in the cytosol, while during starvation TFEB is unphosphorylated and free to move into the nucleus. TFEB phosphorylation is regulated mainly by ERK2 and mTORC1. Notably, mTORC1 and TFEB meet on the lysosomal membrane where mTORC1 phosphorylates TFEB [99,100].

All the reported data suggest that the autophagy picture is still largely incomplete. Beside all the studies concerning autophagosome biogenesis, we still need detailed analysis about the lysosomes biogenesis and their active role in autophagy regulation. Of note is also the analysis of the cytoplasm-to-vacuole targeting pathway, which is the only characterized biosynthetic mechanism that utilizes the Atg proteins [101].

## 6. Conclusions

The autophagy-lysosome system is emerging as a crucial process for controlling muscle health during catabolic conditions. However, autophagy is also required for basal myofiber homeostasis and its deregulation can lead to myofiber degeneration. Defects of the autophagy-lysosome machinery play a role in the pathogenesis of different myopathies and muscular dystrophies, characterized by the presence of protein aggregates, accumulation of abnormal mitochondria and dilations of the sarcoplasmic reticulum. Recent studies revealed that the correct regulation of autophagy is essential for maintaining muscle homeostasis and fulfilling the different needs and requirements of this tissue in response to various stress conditions and physiological stimuli. We therefore need a better knowledge of the signaling pathways that control the autophagy-lysosome system in skeletal muscle, as well as its mutual relationships with the ubiquitin-proteasome system. Although the study of autophagy in skeletal muscle is still at its infancy, works in this field are now rapidly expanding. We can expect that in the next future such studies will allow to get a much deeper understanding of the role of autophagy in healthy and diseased muscle, which in turn are likely to provide valuable advances for human health and new therapeutic venues for muscle diseases. 
